# Prolonged Use of Ertapenem to Treat Infected Diabetic Foot Ulcers

**DOI:** 10.1155/2010/149591

**Published:** 2010-09-28

**Authors:** Ashwin Algudkar, Gidon Ellis, Fareeduddin Ahmad, Hilary Tindall

**Affiliations:** Department of Microbiology, North Middlesex Hospital, Stirling Way, Edmonton, London N18 1QX, UK

## Abstract

We present the case of a diabetic man who was successfully treated with ertapenem for over 4 months for severe infection of his foot ulcers. After initial unsuccessful treatment with empirical intravenous antibiotics, ertapenem was started on microbiology advice and led to a marked improvement in the soft-tissue infection. Ertapenem was continued for a total of 137 days under close clinical and biochemical monitoring and produced a complete resolution of the foot infection. This is the first documented case that we know of in which ertapenem has been safely used for this duration of time.

## 1. Introduction

We present the case of a diabetic man who was successfully treated with ertapenem for over 4 months for severe infection of his foot ulcers. This is the first documented case that we know of in which ertapenem has been safely used for this duration of time.

## 2. Case Presentation

A 69-year-old Caucasian type 2 diabetic man presented to the emergency department with a 2-month history of painful ulcers on his left foot which had been increasing in size. The patient had been diagnosed with type 2 diabetes mellitus 13 years earlier. This was managed with metformin, gliclazide, and rosiglitazone, although the patient admitted to not having taken these medications for over a year. His medical history included hypertension, hypercholesterolaemia, and a previous myocardial infarction. He had a smoking history of 50 pack years, drank approximately 2 units of alcohol per week, and was independent in all activities of daily living. The patient was started on oral flucloxacillin (500 mg qds) and metronidazole (400 mg tds) and discharged from the emergency department to be seen 4 days later in the diabetic foot clinic.

In the clinic, the patient was found to be hypertensive (blood pressure 164/88 mmHg) and tachycardic (pulse 110 beats per minute) with a temperature of 36.4°C and oxygen saturations of 93% on room air. He weighed 64.9 kg and was 1.67 m tall, giving a body mass index of 23.3 kg/m^2^. Inflammatory markers in the blood were raised (normal ranges in brackets): white cell count 16.2 × 10^9^/l (4.0–11.0), neutrophils 12.4 × 10^9^/l (2.0–7.5) and c-reactive protein 46 mg/l (0–6.0). His glycated haemoglobin was noted to be 16.5% (4.1–6.5).

Two ulcers were identified on the left foot: one on the lateral aspect of the heel, measuring 5 × 6 cm and the other on the dorsal aspect of the left hallux, measuring 2 × 2 cm. Both ulcers were necrotic and sloughy and the bone of the hallux was visible through the second ulcer. Peripheral pulses were palpable bilaterally but there was marked peripheral neuropathy of both feet. Systemic examination was unremarkable. 

Swab cultures were taken but only skin flora was isolated (the details of this flora were not disclosed in the microbiology report, however). A specific anaerobic culture was not performed. Blood cultures were also sent, but no bacterial growth was detected, unsurprising given that the patient had already been commenced on oral antibiotics. Radiograph of the left foot demonstrated loss of soft tissues at the ulcer sites but did not show any clear evidence of osteomyelitis. However neither bone scan nor computerised tomography (CT) was performed to confirm the absence of osteomyelitis. 

The patient was admitted to a medical ward and started on empirical intravenous antibiotics: benzylpenicillin 1.2 g qds, flucloxacillin 500 mg qds, and metronidazole 500 mg tds. The ulcers were debrided and daily dressings were applied. The patient also had a short course of maggot therapy to remove the necrotic tissue from the ulcers. Debrided tissue was regrettably not sent for culture as debridement was performed in the podiatry department and not on the ward under medical supervision.

12 days after commencing intravenous antibiotics, oral sodium fusidate 500 mg tds was also started. On the same day, the patient was reviewed by a vascular surgeon with ankle brachial pressure index (ABPI) analysis revealing moderate arterial disease, worse in the left leg compared to the right (ABPI 0.51 in the left leg and 0.56 in the right leg) (ABPI reference ranges: 1.0–1.3 normal, 0.9-1.0 acceptable, 0.8-0.9 mild arterial disease, 0.5–0.8 moderate arterial disease, and <0.5 severe arterial disease). The vascular opinion at this stage was that if the ulcers did not heal with conservative measures then amputation would have to be considered.

3 weeks after the introduction of intravenous antibiotics the appearance of the foot ulcers had not improved. In addition, it appeared that an area of cellulitis was spreading from proximal to the ulcers to the rest of the foot. Blood tests revealed that the patient's white cell count had risen to 19.6 × 10^9^/l and the c-reactive protein had risen to 164 mg/l. However, throughout this time, the patient was afebrile. The microbiology department were consulted and advised stopping the current antibiotics and commencing the patient on intravenous ertapenem 1 g od.

6 weeks after starting ertapenem, the appearance of the left foot was improved. There was significantly less slough at the base of the ulcers, but the heel ulcer was still necrotic. Blood tests revealed that the white cell count had fallen to 14.2 × 10^9^/l and the c-reactive protein had fallen to 9 mg/l. The patient remained afebrile. The decision was made to continue ertapenem therapy as it was felt that the soft-tissue infection had not completely resolved and as bone involvement could not be ruled out, further antimicrobial treatment would be required.

After 3 months of being on ertapenem, the appearances of the foot ulcers had improved further with the inflammatory markers in the blood also falling (white cell count 12.9 × 10^9^/l, c-reactive protein 6 mg/l) and the patient's temperature remaining normal. The patient was being seen in the foot clinic on a weekly basis with debridement of the ulcers taking place when appropriate. The microbiology department felt that ertapenem should be continued as no adverse effects had been encountered and that the soft-tissue infection required further treatment. 

After over 4 months on ertapenem the ulcers had almost completely healed with necrotic tissue no longer visible. The patient's white cell count had fallen to 12.3 × 10^9^/l and the c-reactive protein was 6 mg/l. He had remained afebrile throughout all this time. The patient's glycated haemoglobin was rechecked and had come down to 5.9%. 

Ertapenem was stopped after 4 months and 16 days of therapy (137 days in total). The patient had remained in hospital for the full duration of the ertapenem therapy to ensure compliance with all medications, so that the affected limb was free from weight bearing to allow pressure relief and to permit regular debridement of the ulcers. The patient was discharged home 2 weeks after the cessation of ertapenem. Vascular review before discharge documented that amputation no longer had to be considered at present but that the patient would require close followup in the community and would at some point require vascular intervention.

## 3. Discussion

Foot infections account for approximately 20% of hospital admissions in diabetic patients and up to 25% of all diabetics are expected to develop severe foot problems at some point during their life [1]. Infection is usually a consequence of foot ulcerations which typically result from trauma to a neuropathic foot. Other major risk factors for ulcer formation are structural foot deformity and peripheral vascular disease.

Diabetic foot infections are generally more severe and harder to treat than infections seen in nondiabetic patients. This is due to a number of factors including impaired microvascular circulation, neuropathy, anatomical alterations and impaired immune capacity in diabetic patients.

Ertapenem, a long-acting, 1*β*-methyl parenteral group 1 carbapenem antibiotic with a broad antibacterial spectrum was licensed for clinical practice in Europe and the USA in 2002 [2]. It has a once-daily dosing regimen and is generally well tolerated [3]. Ertapenem is active against gram positive, gram negative, and anaerobic bacteria (Table 1) and has been shown to be effective in skin/soft-tissue infections [4].

Infection of diabetic foot ulcers is usually caused by aerobic gram positive cocci, most commonly *staphylococcus aureus, haemolytic staphylococci* (especially group B), and *coagulase negative staphylococci*. Although monomicrobial infections may occur, it is common for these gram positive cocci to be found along with gram negative bacilli and obligate anaerobes [5, 6]. In this case, the patient did not respond to the initial antibiotic regimen of intravenous benzylpenicillin, flucloxacillin, and metronidazole. This regimen lacks coverage for facultative gram negative rods, and so the failure could have been due to the inadequate coverage for these organisms. 

In this case, the only microbiology isolated was reported as “skin flora”. A multicentre clinical trial on the bacteriology of diabetic foot infections published in 2007 found *staphylococcus epidermidis* in nearly 50% of the coagulase negative staphylococci isolates [7]. Citron et al. comment that *S. epidermis*, often dismissed as a commensal or contaminant, is now increasingly being recognised as a true pathogen in diabetic foot infections. Although the organism is innocuous on intact human skin, it can cause severe infections after it penetrates anatomical barriers partly through the production of proteases, peptidases, biofilms, and surface lipoproteins that promote host tissue adherence [7]. 

Citron et al. go on to state that because specimens from most patients have polymicrobial cultures, empirical therapy should be relatively broad spectrum with either ertapenem or piperacillin-tazobactem being an appropriate empirical single agent (except for methicillin resistant *S. aureus* infections) [7]. 

Ertapenem has a broad spectrum of action, but it does not provide cover against most *enterococcus* or *pseudomonas* species which are often found in diabetic foot infections. This would seemingly put ertapenem at a disadvantage to piperacillin-tazobactam, which does cover these commonly occurring species. However, a large multicentre, double-blinded, randomised clinical trial published in 2005 demonstrated that in diabetic foot infections treatment with a once daily dose of ertapenem produced equivalent clinical outcomes to treatment with a four-time daily dose of piperacillin-tazobactam [8]. 

The recommended duration of antibiotic therapy in diabetic foot infections ranges from 1–4 weeks for soft-tissue infection alone to >6 weeks for unresected osteomyelitis. The manufacturers recommend use of ertapenem for 7–14 days in complicated skin infections without osteomyelitis [9]. Ertapenem was used for a total of 137 days in this case, where the presence of osteomyelitis could not be accurately ruled out. Ertapenem was used for this duration of time as at each stage of review, it was felt that although the foot infection was improving further treatment was needed, and as there were no adverse effects this length of therapy was deemed acceptable by both the microbiology department and the admitting medical team.

Clearly, other factors had an important role to play in the resolution of the diabetic foot infection detailed in this case aside from the antimicrobial treatment. When the patient was initially seen in the diabetic foot clinic, he admitted to not taking his oral hypoglycaemic medications for over one year. Whilst he was an inpatient, these medications were restarted and subsequently led to an improvement in glycaemic control. This is reflected in the fall in glycated haemoglobin from 16.5% on admission to 5.9% on discharge. In addition, the patient was not weight bearing on the affected limb throughout the duration of his treatment which would have helped the healing of the foot ulcers through pressure relief. Also the patient's ulcers were regularly debrided allowing healthy tissue to emerge after nonviable tissue had been resected.

This case demonstrates the safe use of ertapenem for over 4 months in treating infected diabetic foot ulcers. In our case, it was highly effective in treating the patient's infected ulcers when empirical therapies had been unsuccessful. Ertapenem has a once-daily dosing regimen and has shown equivalent results in the treatment of diabetic soft tissue infections to the more widely used drug piperacillin-tazobactem. This case highlights the fact that ertapenem could have a role as a first line intravenous antibiotic in the treatment of diabetic foot infections and be used for long durations when required. The once-daily dosing regimen of ertapenem also makes it a suitable agent for prolonged outpatient or home intravenous antibiotic therapy.

## Figures and Tables

**Table 1:** Microorganisms that ertapenem has in vitro and clinical activity against.

Aerobic and facultative gram-positive microorganisms	Aerobic and facultative gram-negative microorganisms	Anaerobic microorganisms
*Staphylococcus aureus *(methicillin susceptible isolates only)	*Escherichia coli*	*Bacteroides fragilis*
*Streptococcus agalactiae*	*Haemophilus influenzae *(beta-lactamase negative isolates only)	*Bacteroides distasonis*
*Streptococcus pneumoniae *(penicillin susceptible isolates only)	*Klebsiella pneumoniae*	*Bacteroides ovatus*
*Streptococcus pyogenes*		*Bacteroides thetaiotaomicron*
		*Bacteroides uniformis*
	*Moraxella catarrhalis*	*Clostridium clostridioforme*
	*Proteus mirabilis*	*Eubacterium lentum*
		*Peptostreptococcus *species
		*Porphyromonas asaccharolytica*
		*Prevotella bivia*
